# Ocular *Mycobacterium haemophilum* infection originating in the cornea: a case report

**DOI:** 10.1186/s12879-023-08094-2

**Published:** 2023-03-07

**Authors:** Yu-Qiang Zhang, Ting-Ting Xu, Fu-Yan Wang, Shuang Wang, Jun Cheng

**Affiliations:** 1 Qingdao University, Qingdao, China; 2 Eye Institute of Shandong First Medical University, Qingdao Eye Hospital of Shandong First Medical University, Qingdao, China; 3 State Key Laboratory Cultivation Base, Shandong Provincial Key Laboratory of Ophthalmology, Qingdao, China; 4 School of Ophthalmology, Shandong First Medical University, Qingdao, China; 5 Anqiu People’s Hospital, Weifang, China; 6 Institute of Clinical Medicine, Weifang Medical University, No. 7166 Baotong West Street, Weifang, China; 7 Shandong Eye Institute, 5 Yanerdao Road, Qingdao, 266071 China

**Keywords:** *Mycobacterium haemophilum*, Keratitis, Conjunctivitis, High-throughput sequencing, Case report

## Abstract

**Background:**

*Mycobacterium haemophilum* is a slow-growing non-chromogenic nontuberculous *Mycobacterium* species that can cause skin infection or arthritis in an immunocompromised population or in children. Primary infection of the healthy adult cornea is rare. The special requirements for culture make this pathogen difficult to diagnose. The study aims to report the clinical manifestation and treatment process of corneal infection and notify the awareness of *M. Haemophilus* keratitis among clinicians. This is the first case report of primary *M. haemophilum* infection in the cornea of healthy adults reported in the literature.

**Case presentation:**

A 53-year-old healthy goldminer presented with left eye redness and a history of vision loss for four months. The patient was misdiagnosed with herpes simplex keratitis until *M. haemophilum* was detected using high-throughput sequencing. Penetrating keratoplasty was performed, and a large number of mycobacteria were detected by Ziehl-Neelsen staining of the infected tissue. Three months later, the patient developed conjunctival and eyelid skin infections that manifested as caseous necrosis of the conjunctiva and skin nodules. After excision and debridement of the conjunctival lesions and systemic antituberculosis drug treatment for 10 months, the patient was cured.

**Conclusion:**

*M. haemophilum* could cause primary corneal infection in healthy adults, which is an infrequent or rare infection. Owing to the need for special bacterial culture conditions, conventional culture methods do not provide positive results. High-throughput sequencing can rapidly identify the presence of bacteria, which aids in early diagnosis and timely treatment. Prompt surgical intervention is an effective treatment option for severe keratitis. Long-term systemic antimicrobial therapy is crucial.

## Background

Nontuberculous mycobacteria (NTM) are described as Mycobacterium species other than M. tuberculosis. They are aerobic, non-motile, and non-spore-forming bacilli that naturally exist in water, soil, dust, and other natural environments, and can infect humans and animals [1]. NTM invades the human body through the respiratory tract, gastrointestinal tract, and skin, possibly affecting all organs. The most recent clinical reports of NTM ocular infections include keratitis, followed by endophthalmitis, periocular skin infections, scleritis, dacryocystitis and canaliculitis, orbital infections, uveitis, and conjunctivitis [2]. And the most common NTMs associated with keratitis was *Mycobacterium chelonianum* and *Mycobacterium abscessus* [3, 4].

*Mycobacterium haemophilus* (*M. haemophilum*) is a member of the NTM family and belongs to the slow-growing subgroup of *non-photochromogens*; it usually grows at 30–32 °C and requires at least three or–eight weeks to develop small, smooth, flat, colorless colonies in the medium containing hemin or high-valent iron complex [5]. This type of aerobic, non-spore-forming Acid Fast Bacteria (AFB) Stain-positive bacterium is environmentally abundant and primarily found in water [6]. *M. haemophilum* infections primarily affect immunocompromised populations, causing skin and subcutaneous infections, septic arthritis, tenosynovitis, osteomyelitis, pneumonia, brain abscesses, sepsis, and disseminated infections [7].

According to the literature and to the best of our knowledge, keratitis caused by *M. haemophilus* is rare. There were only two reports of *M. haemophilus* keratitis have been published, which reported scleritis and keratitis in a healthy adults [8] and filamentous keratitis and conjunctival lesions in a patient with graft-versus-host disease (GVHD) [9]. Previous reports showed that infections originating from sclera and conjunction, and infections originating from the cornea were not reported.

Here, we report a case of ocular *M. haemophilus* infection originating in the cornea in a healthy adult patient. The patient first developed corneal infiltration and ulceration, followed by caseous necrosis of the conjunctiva and infectious nodules of the eyelid skin after therapeutic keratoplasty. The patient was cured after local debridement and 10 months systemic antibiotic therapy. To our knowledge, this is the first case of healthy adult cornea primary infection caused by *M. haemophilum.*

## Case presentation

**Fig. 1:**
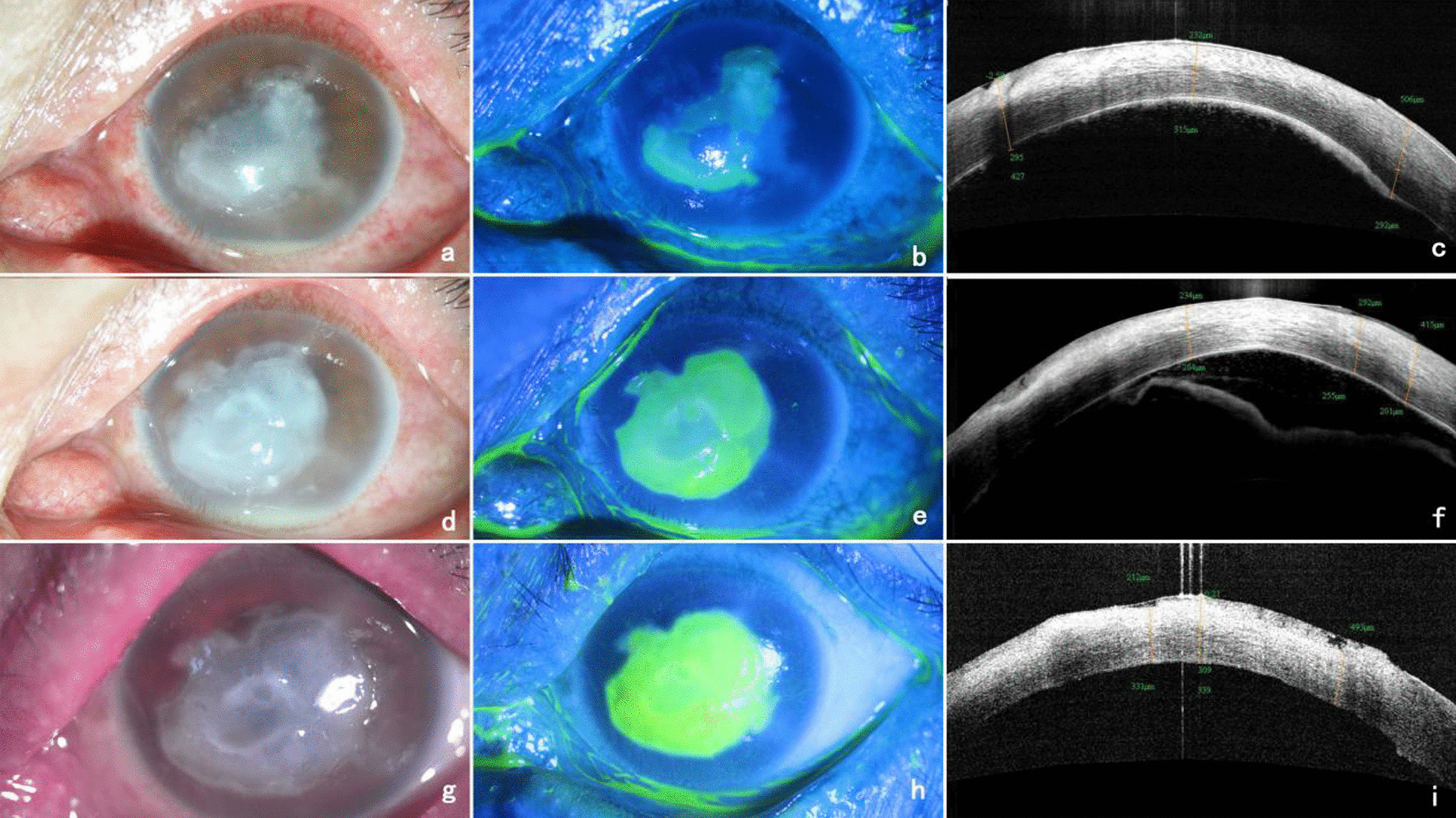
Changes in the patient’s corneal condition before receiving a definitive diagnosis. **a**–**c** show slit-lamp, sodium fluorescein staining and OCT images on admission, respectively; **d**–**f** show slit-lamp, sodium fluorescein staining and OCT images after 5 days of antiviral and glucocorticoid treatment, respectively; **g**–**i** show slit-lamp, sodium fluorescein staining and OCT images after 7 days of antiviral and hormonal treatment, respectively

**Fig. 2:**
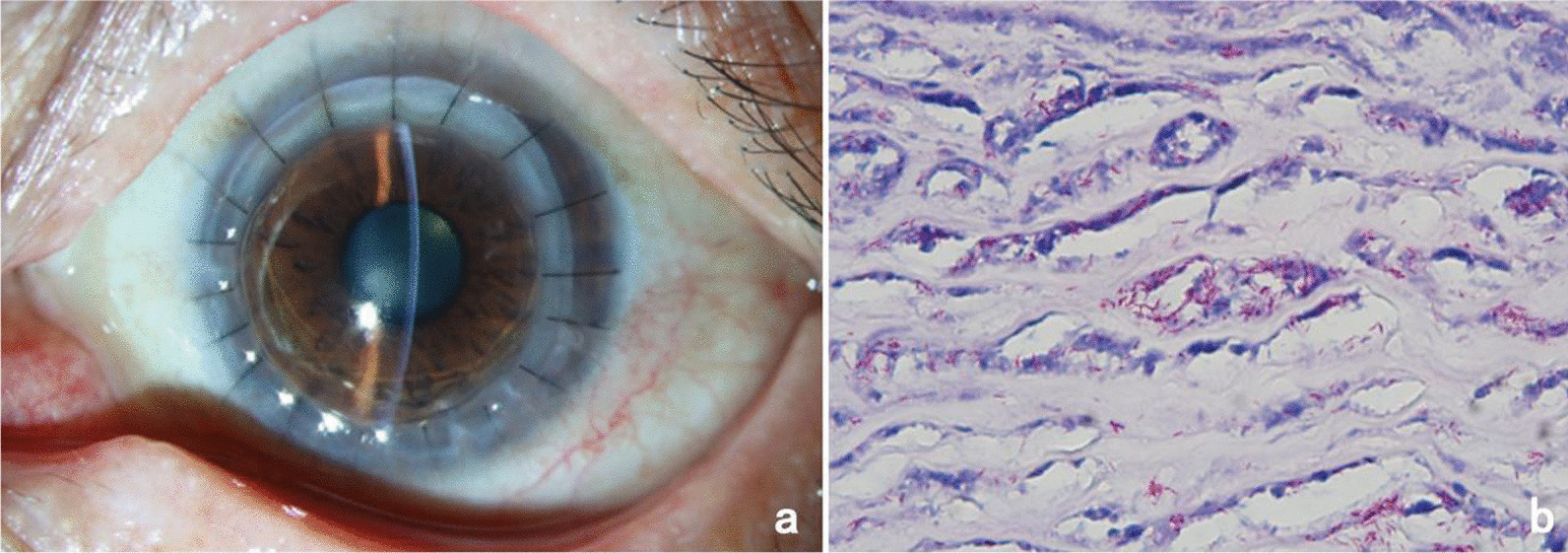
**a** shows slit-lamp examination images 2 months after TPK; **b** shows Ziehl-Neelsen staining of the diseased cornea with a large number of acid-fast bacilli detected in the stroma

**Fig. 3:**
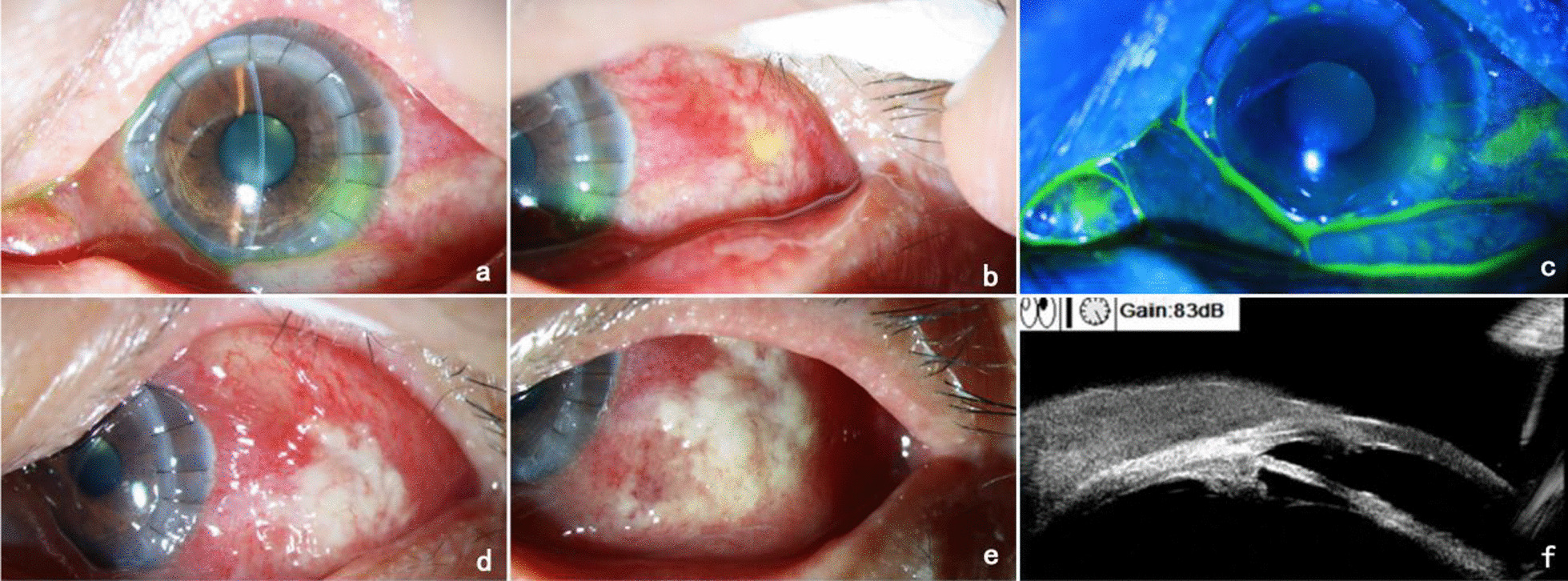
Presentation of the conjunctiva 3 months after TPK treatment for *M. haemophilus* infection. **a** shows a transparent corneal graft with an epithelial defect on the 4-o’clock receptive bed; **b** shows temporal conjunctival congestion and a purulent spot; **c** shows a sodium fluorescein staining image; **d** shows caseous necrosis of the temporal conjunctiva after 2 days; **e** shows an increased extent of caseous necrosis after 3 days; **f** shows thickening of the temporal conjunctiva detected by UBM

**Fig. 4:**
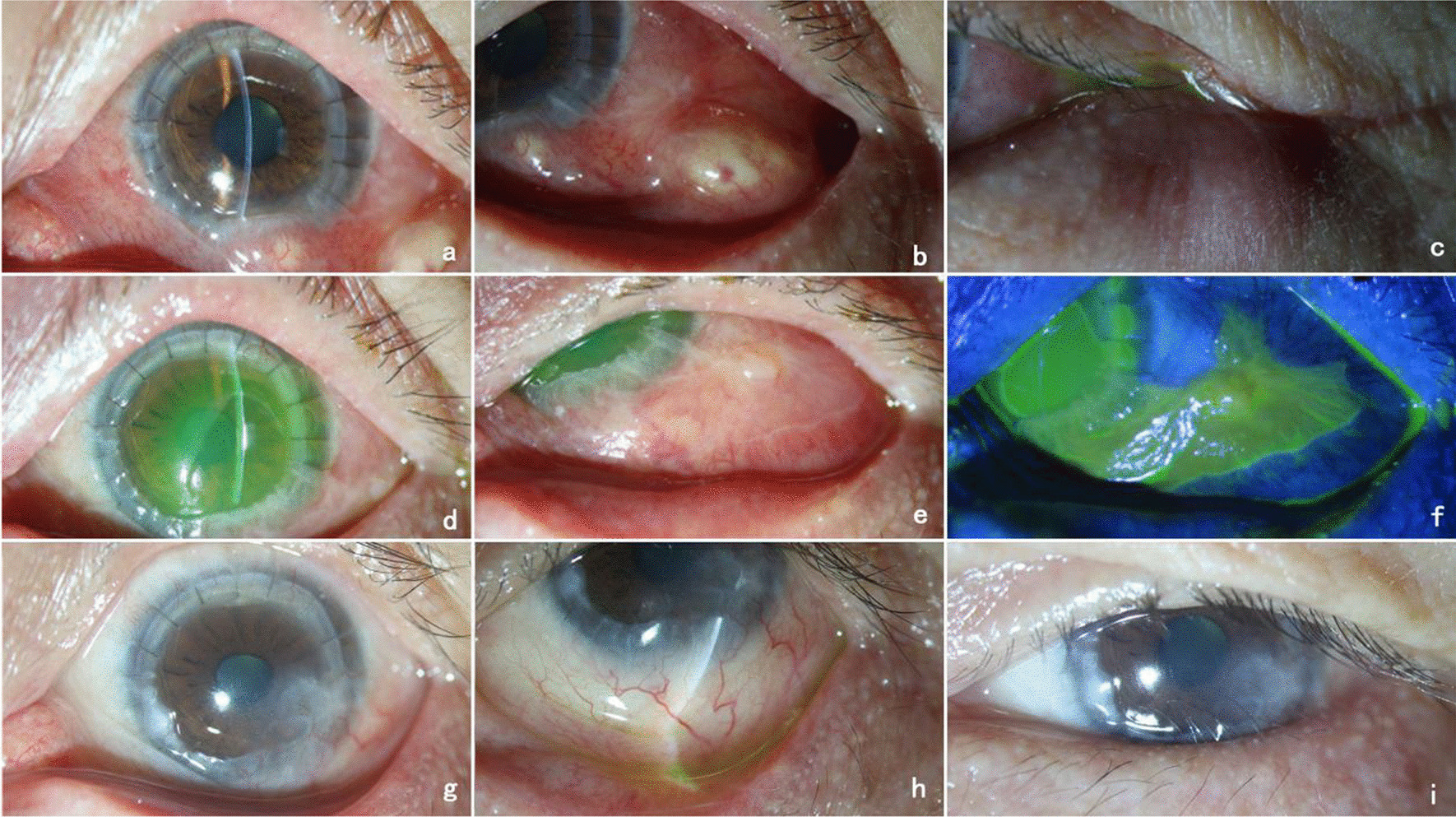
Changes in the patient’s condition after systemic antibiotics. **a**, **b** show abscesses under the lower and inferior temporal conjunctiva after 1 month of systemic antibiotics; c shows nodules on the lateral skin of the lower lid after 1 month of systemic antibiotics; **d**–**f** show that subconjunctival abscesses resolved after 3 months of systemic antibiotics, but large corneal and conjunctival epithelial defects were present; **g**–**i** show the status of the cornea, conjunctiva and eyelids after 7 months of systemic antibiotics

The patient was a 53-year-old Chinese male employed as a goldminer. He presented to our hospital with complaints of uninduced vision loss, redness, and irritation in the left eye for 4 months, which had aggravated in the past week. He denied any history of systemic disease, ocular trauma, or surgery. Prior to visiting our hospital, he was diagnosed with herpes simplex keratitis (HSK) at a local hospital and received topical antiviral and glucocorticoid therapy. The initial examination revealed a visual acuity of hand motion (HM)/10 cm and an intraocular pressure of 11 mmHg in the left eye. Slit-lamp examination of the left eye showed mixed hyperemia, and a large area of irregular matrix turbidity infiltration and irregular defects of the epithelium were observed in the cornea with a 1.5 mm hypopyon (Fig. 1a, b). Confocal microscopy showed a large number of inflammatory cells infiltrating the stroma. Optical coherence tomography (OCT) showed that the corneal stroma was infiltrated and opaque, and a large number of exudates were attached to the endothelium (Fig. 1c). Corneal scrapings were examined with Gram staining and Potassium hydroxide (KOH) wet film, but the test result was negative. The right eye was normal. The patient was suspected to have HSK combined with bacterial infection and was treated with ganciclovir, gatifloxacin, and tobramycin eye drops. After 3 days of treatment, the patient showed no improvement, and tobramycin and dexamethasone eye drops were administered. After the addition of glucocorticoids, inflammation in the anterior chamber was alleviated, but the corneal lesions continued to expand (Fig. 1d–i). To further identify the pathogen, we used a corneal scraper for high-throughput sequencing. The results identified *M. haemophilus*, with 17,608 sequences with a relative abundance of 97.8%. The patient was diagnosed with *M. haemophilus* keratitis, received systemic amikacin with a dosage of 750 mg / day, topical 2% amikacin eye drops once per hour, rifampicin eye drops once per hour, and moxifloxacin eye drops 4 times a day, and underwent therapeutic penetrating keratoplasty (TPK). The cornea collected from the operation was analyzed by Ziehl-Neelsen staining, and a large number of mycobacteria were observed (Fig. 2b). Two months after surgery, the patient’s visual acuity recovered to 0.4 (Fig. 2a). However, 3 months after the operation, the patient returned to the clinic complaining of redness and swelling in the left eye for nearly 4 days. A slit-lamp examination showed that the corneal graft was transparent, and the corneal epithelial defect at the recipient bed was at the 4 o’clock position. The temporal bulbar conjunctiva was hyperemic and edematous, and white pus was observed locally (Fig. 3a–c). A conjunctival *M. haemophilus* infection was suspected. On the following days, the patient deteriorated rapidly, with caseous necrosis of the conjunctiva that rapidly expanded (Fig. 3d, e). Ultrasound biomicroscopy (UBM) revealed conjunctival edema and thickening (Fig. 3f). Debridement was performed in the area of conjunctival necrosis, and acid-fast staining of the specimens revealed a large number of mycobacteria. Histopathological examination of the infected conjunctival tissue showed granulomatous findings. High-throughput sequencing confirmed *M. haemophilus* infection (sequence 135,965, relative abundance 99.58%). Systemic examination revealed no infection in other parts of the body, and laboratory results for anti-HIV, VDRL, TPHA, IgG, IgM, IgA, rheumatoid factor, antinuclear antibodies, and Toxoplasma serotry were all negative. The patient received systemic rifampin (600 mg / day), moxifloxacin (400 mg / day), clarithromycin (750 mg / day), and amikacin (750 mg / day) in addition to topical 2% amikacin (once per hour), moxifloxacin (4 times a day), and rifampicin (once per hour). Systemic dose had been maintained at the original dose, topical dose has been changed to once every 2 h after one month of use, and changed to four times a day after three months, and then maintenance treatment. One month later, the patient’s original conjunctival ulcer was repaired, but abscesses in the inferior and temporal subconjunctiva developed, and a nodule appeared on the outer skin of the lower eyelid (Fig. 4a–c). After three months of treatment, the conjunctival abscess and skin nodule resolved, and the corneal stroma remained transparent despite the development of a large defect in the conjunctival and corneal epithelia (Fig. 4d–f). After 7 months of treatment, the patient’s condition was controlled, the corneal graft was mostly transparent, the visual acuity was 0.3, the conjunctival lesions subsided, and a small scar and mild lower eyelid inversion trichiasis remained (Fig. 4g–i). Systemic antibiotic therapy was continued for 10 months, with no relapse one year after discontinuation.

## Discussion and conclusions

Most infections caused by *M. haemophilus* occur in immunocompromised populations (e.g., HIV-infected patients, organ transplant recipients, patients with autoimmune diseases, and those with IgA deficiency), patients with uncontrolled diabetes, and young children [8–11]. Immunocompetent individuals are rarely infected by *M. haemophilus*. However, in recent years, patients have been reported to be infected by tattoos, eyebrow tattooing, and acupuncture [12, 13], and trauma can lead to *M. hemophilus* infection. In addition, multiple ocular surgeries may cause ocular *M. haemophilus* infections [14].

The patient reported herein was a healthy adult with no medical history. All systemic examinations performed after eye infection were normal, and no infections were found in other parts of the body. The patient, a gold miner who had worked in a wet environment for a long time, may have been exposed to sewage containing *M. haemophilus*. Unnoticed or undetected trauma, such as the casual eye injury, may have likely transmitted the bacteria and cause his illness. This case differs from other reported ocular infections, because the infection was initially located in the cornea. After therapeutic keratoplasty, we thought that the lesions had been removed and the infection had been cured; however, the conjunctival infection had been arised by local spread. The conjunctival infection was speculated to have originated from the direct spread of bacteria, and the eyelid infection was due to continuous or lymphatic dissemination. In addition, local use of corticosteroids may also accelerate the spread of infection. Pisitpayat et al. [8] reported a case of scleritis and keratitis caused by *M. haemophilus* in a healthy adult, similar to our case. However, the case reported by Pisitpayat et al. may have been an initial sclera-infection. Therefore, our case is the first reported corneal-initial *M. haemophilus* infection that showed no evidence of any systemic association.

The lack of specific signs and the very slow onset of keratitis caused by *M. haemophilus*, together with the specific culture requirements of this mycobacterium, makes the diagnosis of this disease very difficult. This slow-growing mycobacterium usually requires eight weeks to grow in media at 30–32 °C, which is lower than the temperature required by other NTMs (35–37 °C). In addition, it requires the addition of hemin or a high-valent iron complex in the media, hence the name “Haemophilus” [6]. Inappropriate culture techniques may lead to false negative results. Ziehl-Neelsen staining of corneal scrapings and tissue specimens is a diagnostic method for mycobacterial infection. However, we did not consider a rare infection, such as mycobacteria, in our patient, and consequently did not adopt this test. High-throughput DNA sequencing was used to confirm the diagnosis. PCR detection is also a reliable and widely used method for the rapid diagnosis of pathogens.

The ophthalmologist had no experience with the clinical features of *M. hemophilus* keratitis. The patients with keratitis reported by Pisitpayat et al. [8] initially presented with radial keratitis, which was misdiagnosed as viral keratitis, and were administered antiviral therapy and long-term glucocorticoid therapy. This approach can control symptoms; however, the condition worsens dramatically. Similarly, the patient described herein was initially misdiagnosed with HSK at a local hospital and was misdiagnosed with HSK after visiting our hospital because the corneal lesions showed geographic epithelial defects and infiltration. After antiviral and glucocorticoid therapy, the patient’s inflammatory response was alleviated, but the range of the lesions gradually expanded. A study by Ford et al. [3] found that 80% of patients with NTM keratitis who received topical glucocorticoids did not respond to antibiotics. Glucocorticoid therapy reduces local host immune defenses and contributes to the development and progression of NTM keratitis.

Histopathologically, the most commonly reported cutaneous manifestations of *M. haemophilus* infection are mixed granulomas and a purulent reaction with various histological changes, including panniculitis, ulcer necrosis, abscess formation, lichenoid interface dermatitis, and lymphocyte vasculitis [15]. The patient described herein presented with caseous necrosis in the conjunctival lesions, and histopathological examination also showed granulomatous findings. Therefore, mycobacterial infections should be suspected when similar findings are observed.

A consensus on the treatment modality and duration of *M. haemophilus* infection is currently lacking. Moxifloxacin, clarithromycin, azithromycin, rifampicin, levofloxacin, and amikacin are commonly used antibiotics reported in the literature, and three to four types of antibiotics should be systemically used in combination to adequately control infection. Nookeu et al. [16] believed that the treatment of *M. haemophilus* infection should last for 3–12 months and should be tailored to the severity of the disease and immunocompromised conditions. Recurrence cases have also been reported. Lindeboom et al. [7] reported a recurrence rate of 4% in cases of *M. hemophilus* infection, whereas Nookeu et al. [16] reported a recurrence rate of 14%. Therefore, the patient’s status should be carefully monitored after treatment discontinuation. Prompt surgical resection of the lesion is considered an effective treatment method. For patients with severe corneal infections, therapeutic keratoplasty can be considered; however, if the corneal lesions are not completely removed, the risk of recurrence is high. Infections in the peripheral cornea can progress to the limbus, leading to infections of the conjunctiva, sclera, and even endophthalmitis. Conjunctival and skin infections can be treated with debridement.

In conclusion, *M. haemophilus* could cause primary infection of the cornea in healthy adults and spread to other tissues of the eye by direct and hematogenous spread. High-throughput DNA sequencing can quickly provide an accurate diagnosis. Therapeutic keratoplasty and debridement of necrotic tissue are effective treatments for severe infection. Long-term and systemic administration of sensitive antibiotics is a necessary strategy for a radical cure. The patient’s status should be monitored on a long-term basis for the timely detection of recurrent infections. Once diagnosed, glucocorticoid use should be stopped to avoid the spread of infection.

## Data Availability

The data that support the findings of this study are available from the corresponding author (CJ) upon reasonable request.

## Abbreviations

M. *haemophilum*: *Mycobacterium haemophilum*
NTM: Nontuberculous mycobacteria
GVHD: Graft-versus-host disease
HSK: Herpes simplex keratitis
HM: Hand motion
OCT: Optical coherence tomography
KOH: Potassium hydroxide
TPK: Therapeutic penetrating keratoplasty
UBM: Ultrasound biomicroscopy
